# PcoB is a defense outer membrane protein that facilitates cellular uptake of copper

**DOI:** 10.1002/pro.4364

**Published:** 2022-06-21

**Authors:** Ping Li, Niloofar Nayeri, Kamil Górecki, Eva Ramos Becares, Kaituo Wang, Dhani Ram Mahato, Magnus Andersson, Sameera S. Abeyrathna, Karin Lindkvist‐Petersson, Gabriele Meloni, Julie Winkel Missel, Pontus Gourdon

**Affiliations:** ^1^ Department of Experimental Medical Science Lund University Lund Sweden; ^2^ Department of Biomedical Sciences University of Copenhagen Copenhagen Denmark; ^3^ Department of Chemistry Umeå University Umeå Sweden; ^4^ Department of Chemistry and Biochemistry The University of Texas at Dallas Richardson Texas USA

**Keywords:** gut microbiota, outer membrane protein structure, PcoB

## Abstract

Copper (Cu) is one of the most abundant trace metals in all organisms, involved in a plethora of cellular processes. Yet elevated concentrations of the element are harmful, and interestingly prokaryotes are more sensitive for environmental Cu stress than humans. Various transport systems are present to maintain intracellular Cu homeostasis, including the prokaryotic plasmid‐encoded multiprotein *pco* operon, which is generally assigned as a defense mechanism against elevated Cu concentrations. Here we structurally and functionally characterize the outer membrane component of the Pco system, PcoB, recovering a 2.0 Å structure, revealing a classical β‐barrel architecture. Unexpectedly, we identify a large opening on the extracellular side, linked to a considerably electronegative funnel that becomes narrower towards the periplasm, defining an ion‐conducting pathway as also supported by metal binding quantification via inductively coupled plasma mass spectrometry and molecular dynamics (MD) simulations. However, the structure is partially obstructed towards the periplasmic side, and yet flux is permitted in the presence of a Cu gradient as shown by functional characterization in vitro. Complementary in vivo experiments demonstrate that isolated PcoB confers increased sensitivity towards Cu. Aggregated, our findings indicate that PcoB serves to permit Cu import. Thus, it is possible the Pco system physiologically accumulates Cu in the periplasm as a part of an unorthodox defense mechanism against metal stress. These results point to a previously unrecognized principle of maintaining Cu homeostasis and may as such also assist in the understanding and in efforts towards combatting bacterial infections of Pco‐harboring pathogens.

## Abstract 2

PDB Code(s): 7PGE;

## 1 INTRODUCTION

**FIGURE 1:**
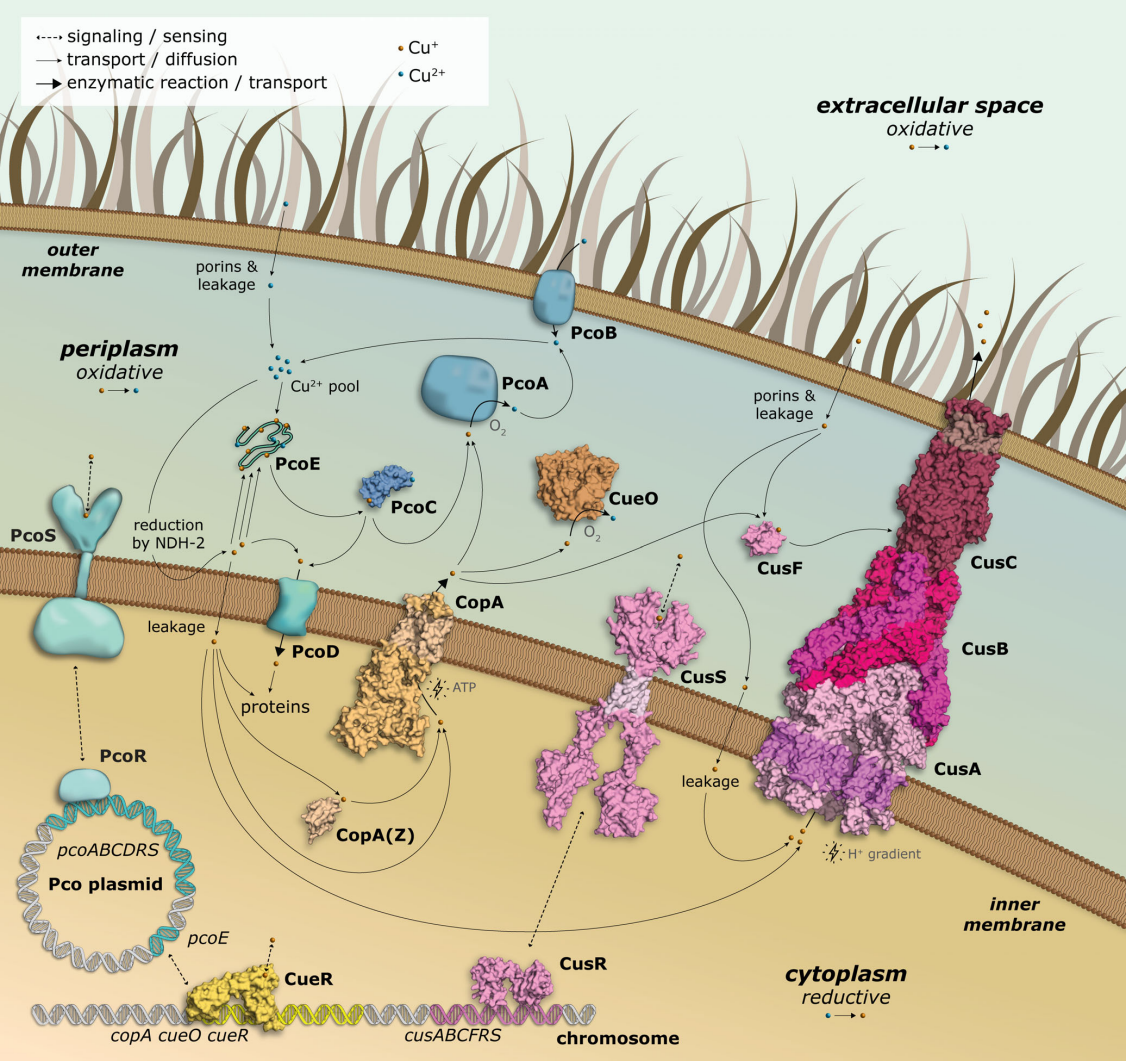
Overview of copper homeostasis proteins in gram negative bacteria. The Cue/Cop system (in yellow) is responsible for copper detoxification through removal from the cytoplasm via CopA and periplasmic oxidation via CueO as regulated by CueR. At higher copper concentrations or anaerobic conditions, the Cus assembly (magenta) provides export from the cytoplasm via CusA, and from the periplasm via CusF, immediately to the extracellular environment as allowed by CusB and CusC and with the expression regulated by CusR and CusS. The plasmid born Pco cluster (cyan) likely has a complementary role, harboring an inner and outer membrane component, PcoD and PcoB, respectively, and the periplasmic oxidase PcoA as well as the copper‐binding PcoC and PcoE proteins, as regulated by PcoR and PcoS. The structures were generated from PDB entries: 3RFU (CopA), 1KV7 (CueO), 1Q05 (CueR), 3NE5 (CusBA), 3PIK (CusC), 2VB2 (CusF), 5KU5 (CusS), 1LYQ (PcoC). Fragments of CusS, CusR and CopA(Z) are structure predictions from AlphaFold Protein Structure Database.^30^, ^31^ PcoE is shown as a polypeptide chain rather than a surface representation due to its disordered nature

Copper (Cu) is a transition metal essential for virtually all organisms, for example serving as a co‐factor for a number of enzymes involved in redox reactions.^1^ However, elevated Cu levels is associated with mismetallation and damage to proteins and cells, and catalyze toxic reactive oxygen and nitrogen species production via redox cycling.^2^ Strikingly, mammals are frequently more tolerant to increased Cu levels in the surroundings than prokaryotic counterparts.^3^ Organisms have developed mechanisms for tight regulation of the Cu levels.^4^ The significance of maintained Cu homeostasis is underscored by the many different protein networks linked to this process. In *Escherichia coli* (*E. coli*), the cytoplasm is maintained devoid of free Cu via its export mediated by the Cue/Cop system, regulated by the transcription factor CueR^5^ (Figure 1, yellow^6^, ^7^). CopA, an inner membrane P‐type ATPase, extrudes Cu^+^ ions to the periplasm,^8^ where it is oxidized to less toxic Cu^2+^ by CueO, a multicopper oxidase.^9^ Once this response is overwhelmed or under anaerobic conditions, when the CueO oxidase is inactive, the Cus‐mediated Cu export assembly is activated (Figure 1, magenta^10^). Cus connects the inner and outer membranes, spanning the entire periplasm^10^ through three proteins CusCBA, and is energized by the tripartite resistance‐nodulation‐cell division CusA component, collectively providing capacity to export Cu^+^ from the cytoplasm directly out of the cell.^11^ Additionally, CusF, a periplasmic Cu‐sequestering protein, delivers the metal directly to CusB for efflux.^12^ The expression of the Cus constituents is regulated by CusRS.^13^, ^14^ Considering the Cus system limitations, complementary Cu homeostasis proteins exist, most notably the plasmid‐born Cu resistance Pco system (Figure 1, cyan). This operon was first detected in *E. coli* from the gut flora of pigs fed a high‐Cu diet^15^; Cu in combination with antibiotics have been used as growth promotor in pig diets for at least 45 years.^16^

The Pco proteins have been shown to enable bacteria to survive at higher Cu concentrations, although *E. coli* strains lacking the genes accumulate less Cu in the periplasm and exhibit higher Cu efflux.^15^, ^17^ Underscoring the significance of the Pco assembly, homologous proteins are frequently present on chromosomes or plasmids of other bacteria, where they also have been linked to increased Cu tolerance.^15^, ^18^ Nonetheless, there is growing evidence congruent with the Pco proteins also being involved in Cu uptake,^17^, ^19^ seemingly in conflict with the observed role for Cu defense. Thus, even the physiological role of the Pco proteins for Cu homeostasis in bacteria remains enigmatic.

The pco gene cluster in *E. coli* encompasses seven genes, pcoABCDRSE.^20^ The PcoRS is a two‐component regulatory system, analogous to CusRS, sensing the periplasmic Cu concentrations.^20^ PcoE resides in the periplasm and binds Cu, predicted to serve as a “molecular sponge”, thereby decreasing the free Cu concentration in the compartment between the two cell membranes.^21^ PcoD represents an inner membrane protein, and the function is likely tightly linked to periplasmic PcoC as they often exist as a fusion protein. For example, the *Bacillus subtilis* single protein YcnJ shares high‐sequence homology to the two PcoCD components. Deletion of YcnJ is associated with impaired growth in low‐Cu media suggesting a putative role in Cu acquisition,^17^ while expression of PcoABD leads to Cu hypersensitivity in the absence of PcoC.^22^

Similarly to PcoCD, PcoAB have been proposed to work together as the primary actors in pco‐dependent Cu resistance.^23^ While PcoA is a periplasmic multicopper oxidase, distantly related to CueO, PcoB resides in the outer membrane and has an elusive function.^20^ PcoB has been suggested to prevent Cu uptake from the cellular outside,^24^ however, since CopA appears necessary for Pco‐dependent Cu resistance, PcoB is generally believed to be a Cu‐specific transport protein, acting in concert with PcoA.^24^ Homologues of PcoAB are regularly encoded in close proximity in gram‐negative bacteria, and whereas PcoA is sometimes found alone, PcoB is always accompanied by PcoA, suggesting the interaction between the two and that PcoB requires PcoA for the Cu‐transport function.^25^ For instance, expression of PcoB alone in the absence of PcoA in a ∆pcoAB *Caulobacter crescentus* strain did not rescue the Cu‐sensitive phenotype.^26^ However, PcoC was also shown to be needed for full resistance of the Pco system, and to interact with PcoA, possibly serving as a periplasmic Cu‐chaperone.^27^ Collectively, the molecular details of the function and the regulation of the Pco system remains elusive.

In this work, we set out to elucidate the physiological role and functional properties of PcoB. We determine the 3D structure and characterize the protein function in vitro and in vivo. Our findings shed fundamentally new light on the role of the Pco system in Cu homeostasis.

## 2 RESULTS AND DISCUSSION

### 2.1 Pco confers cell survival at elevated Cu levels but isolated PcoB sensitizes to Cu stress

**FIGURE 2:**
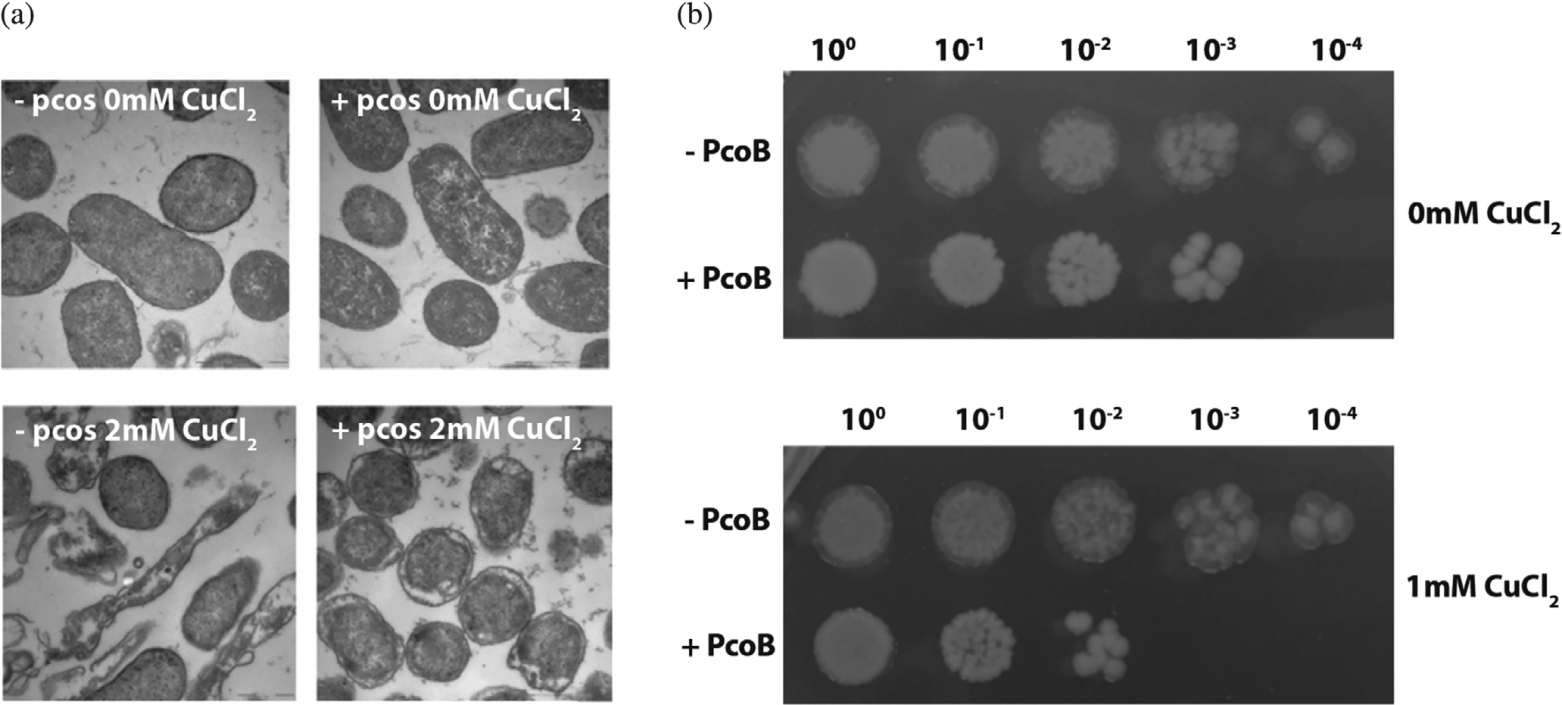
In vivo experiments support a protective role of the Pco system while isolated PcoB increases copper sensitivity. (a) Introduction of the Pco gene cluster (right panels, labelled with +) rescues *Escherichia coli* (*E. coli*) viability at elevated (2 mM) copper concentrations (lower panels), as compared to cells lacking the Pco system (left panels) (b). Comparison of the growth of *E. coli* cells with or without isolated PcoB at low (no supplementation) and high (1 mM) copper concentrations, respectively, suggests PcoB alone increases the copper susceptivity of cells

To further dissect the physiological role of the Pco system, we first compared *E. coli* cells transfected with a vector harboring the operon or a similar control vector lacking the Pco components. Using electron microscopy, the strains were studied at low‐ and high‐Cu levels, respectively. While in a Cu‐low environment both systems emerged as healthy intact cells (Figure 2a, upper panels), the strain without the Pco system appeared impeded under Cu stress (Figure 2a, left bottom panel). In contrast, cell viability was maintained at high‐Cu concentrations for the cells with the pco operon (Figure 2a, right bottom panel), yet displaying a somewhat different morphology compared to the cells proliferated in Cu‐depleted conditions. Specifically, the Pco harboring cells preserve the overall shape and display separation of the cell interior from the plasma membrane, while many cells without the Pco system have adopted an elongated character, with certain cells even appearing disrupted. Collectively, these findings suggest the Pco proteins serve a role for cell survival at elevated Cu concentrations, congruent with the bulk of the literature on the system. While these observations substantiate a role for the entire Pco operon in promoting Cu resistance, the role of individual proteins within the operon cannot be readily dissected with the data. In this context, the observation of putative Pco‐mediated import is puzzling,^28^ and thus highlights the enigmatic specific roles of each Pco protein present in outer membrane (PcoB), the periplasmic space (PcoA, PcoC and PcoE) and the inner membrane, (PcoD). In particular, the role of PcoB, which represents the first component facing cellular exposure to extracellular Cu and the first line of defense upon Cu stress, remains elusive. Is PcoB facilitating import or export of Cu?

Next, to shed further light on the specific function of PcoB, we investigated the protein outside of the context of the other Pco proteins. Surprisingly, *E. coli* cells expressing PcoB showed increased sensitivity towards Cu compared to cells without the protein in a growth assay (Figure 2b). Thus, PcoB appears to serve as a Cu importer, alternatively other components of the Pco system are necessary for the system to operate as an exporter, as CusA is required for CusC.^29^

### 2.2 The molecular structure of PcoB

**FIGURE 3:**
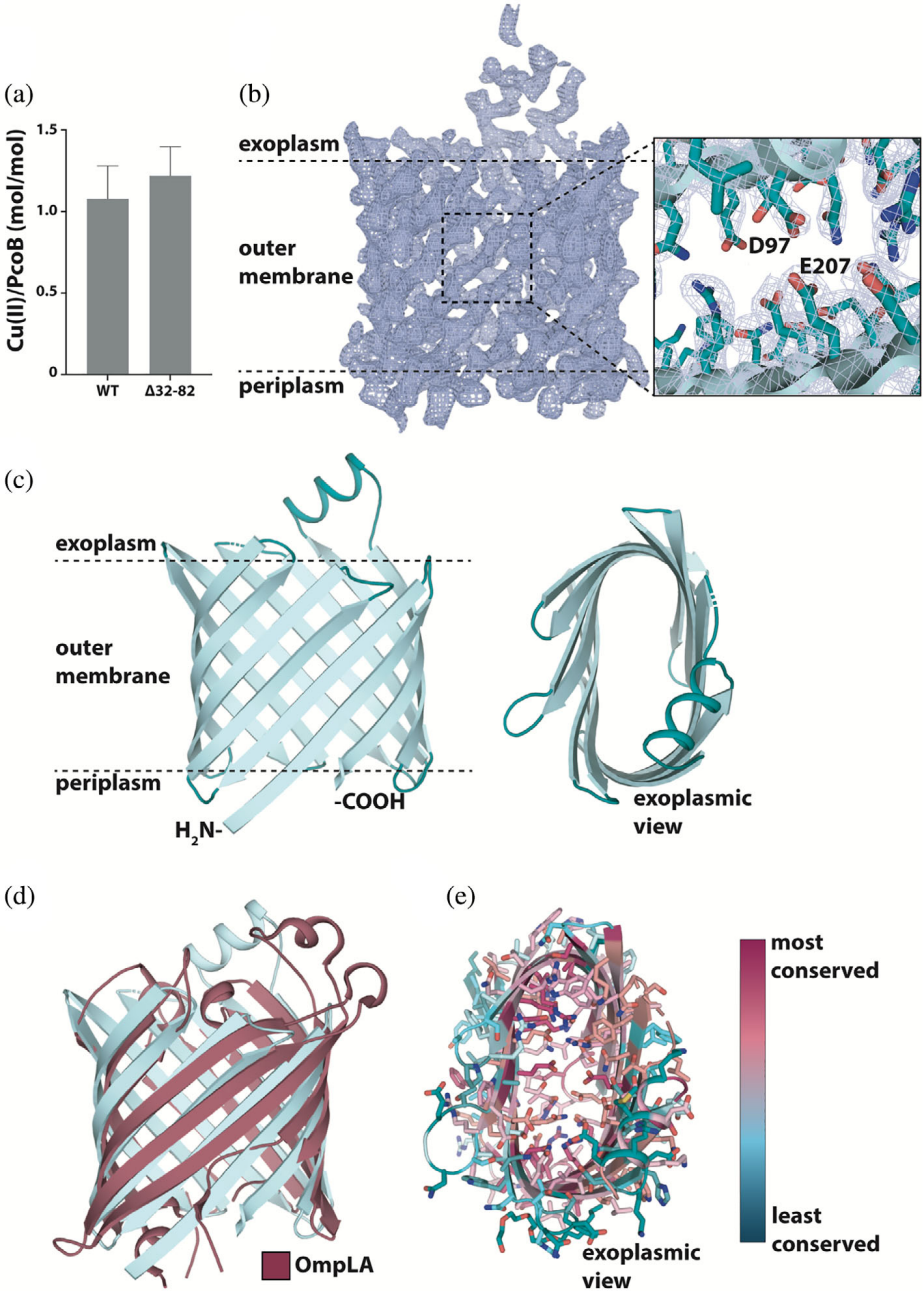
The structure of PcoB. (a) Copper binding stoichiometry as determined using ICP‐MS measurements of full‐length (wild type) and N‐terminally truncated PcoB, respectively. A single Cu^2+^ ion binds per PcoB molecule. Data denotes three independent experiments measurements and error bars represent SD. (b) Final 2Fo‐Fc electron density (blue mesh, *σ* = 1.0) of PcoB derived from the 2.0 Å native data. The close view demonstrates the general quality of the map and the D97‐E207 interaction in the pore region. (c) The overall architecture of PcoB (in cyan) consisting of 12 beta‐sheets that span the outer membrane. The PcoB barrel (cyan) is flattened through interactions between residue of opposite sides of the inside of the barrel, see also panel D. (d) Overlay with structurally reminiscent protein OmpLA (dark red, PDB‐ID: 1QD6). (e) Analysis of the conservation of PcoB as determined using ConSurf.^34^ The analysis shows a spectrum ranging from low (cyan) to high (magenta) conservation, as also depicted by the color bar. Internal residues are generally more conserved than membrane facing residues

To elucidate underlying mechanism behind the observed contradicting physiological responses and define the PcoB directionality of the metal cargo (Cu^2+^) transport, we sought out to obtain high‐resolution structural information of PcoB. The protein was overproduced, extracted from *E. coli* outer membranes and purified to near homogeneity, eluting at a size‐exclusion chromatography (SEC) retention volume that indicated monomeric particle distribution. However, efforts to crystallize full‐length PcoB, PcoB_FL_, were fruitless. In contrast, N‐terminally truncated PcoB, PcoB_Δ27−81_ (Figure S1A), a form that does not alter the Cu‐binding properties (Figure 3a), successfully yielded crystals in the presence of the detergent C_8_E_4_, which diffracted to 2.0 Å. Nevertheless, a reliable molecular replacement solution was not identified. Instead, the structure was determined using single‐wavelength anomalous diffraction (SAD) based on SeMet‐data (Table 1) and refined to R/Rfree = 0.22/0.26. Overall, the generated electron density maps are of high quality, permitting assignment of individual sidechains (Figure 3b), with the exception for the N‐terminus, which is predicted to be highly flexible according to an AlphaFold model^30^, ^31^ (Figure S1B), and a single loop (residues G82‐A89 and D230‐R238), which appears disordered (Figure S2).

**TABLE 1:** Crystallographic table of PcoBΔ32‐82 for both native and SeMet crystals

	*Native*	*SeMet*
*Data*		
Collection wavelength	1	0.979587
Resolution range	43.53–2.0 (2.072–2.0)	46.25–2.62 (2.74–2.62)
Space group	C 2 2 2_1_	P 4_1_ 2_1_ 2
Unit cell	65.49 Å 75.54 Å 91.51 Å 90° 90° 90°	64.76 Å 64.76 Å 198.20 Å 90° 90° 90°
Total reflections	103,621 (10567)	343,385 (41634)
Unique reflections	15,638 (1528)	13,396 (1554)
Multiplicity	6.6 (6.9)	25.6 (26.8)
Completeness (%)	99.72 (99.74)	99.7 (98.2)
Mean I/sigma(I)	16.51 (2.11)	19.0 (0.8)
R‐merge	0.06975 (0.8315)	0.103 (5.697)
CC1/2	0.999 (0.8)	1.0 (0.676)
CC*	1 (0.934)	1.0 (1.0)
Anomalous completeness		99.7(98.1)
Anomalous multiplicity		14.3(14.4)
*Refinement*		
Reflections used in refinement	15,631 (1525)	
R‐work	0.2205	
R‐free	0.2533	
Number of nonhydrogen atoms	1,686	
Macromolecules	1,599	
Ligands	40	
Solvent	47	
RMS (bonds)	0.008	
RMS (angles)	1.08	
Average B‐factor	48.02	
Macromolecules	47.97	
Ligands	51.31	
Solvent	46.87	

**FIGURE 4:**
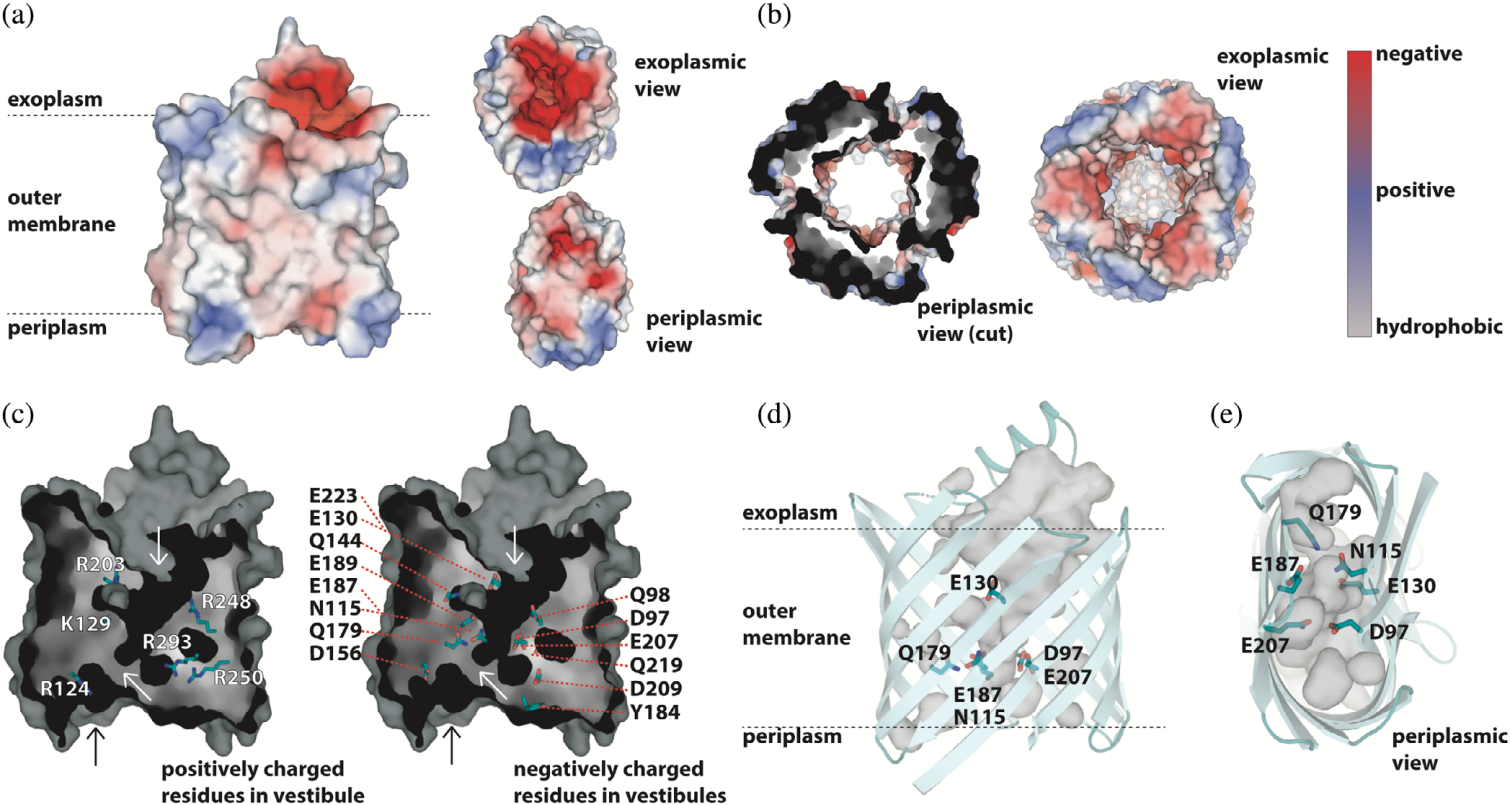
The pore. (a) Surface charge representation of PcoB as observed perpendicular to the membrane, complemented by views from the outside of the cell and from the periplasm, respectively. Electrostatics are represented as positive (blue), negative (red) and neutral (grey/white) charges. (b) Electrostatic representation of CusC (PDB‐ID: 3PIK) using the same color representation as in panel A. The periplasmic view is cut at the outer membrane interface, removing the soluble domain. (c) Surface representation of the pore and internal cavities combined with residues of positive and negative charge, respectively. The top white arrow points to the funnel, the middle white arrow points to the internal constriction site. The black arrow indicates the vestibule observed in panel A bottom view. (d) Side‐view perpendicular to the membrane. Important residues for the here proposed copper conducting pathway are shown. (e) Periplasmic view (same orientation as the periplasmic view in panel A), showing the same features as panel D

The structure discloses a classical β‐barrel (Figure 3c), formed by 12 antiparallel strands that span the outer membrane in vivo. The termini are located at the same end of the barrel, in agreement with periplasmic localization, as established by numerous studies on proteins with a barrel topology.^32^ The strand linking loops are generally short, except for a loop with a helix insertion, stretching into the extracellular space (Figure 3c). Notably, the overall fold is reminiscent to that of the outer membrane phospholipase A1 (OmpLA), despite low sequence similarity and unique molecular functions (Figure 3d). Indeed, OmpLA contains extensive loops blocking the orifice, and a hydrophobic interior,^33^ characteristics that separate the protein types. However, the architecture of the PcoB barrel is flattened as a consequence of intra‐barrel contacts between charged amino acids, thus restricting the width (Figure 3c,e and Figure 4d,e), whereas these interactions of OmpLA are caused by hydrophobicity. The majority of the sheets consist of alternating hydrophobic and hydrophilic residues, facing the surrounding environment, and pointing into the channel, respectively, as typical for outer membrane proteins. Equivalently, sequence (Clustal Ω) and surface (ConSurf)^34^ conservation analyses suggest residues with sidechains located inside the β‐barrel to be relatively conserved, while membrane exposed amino acids are not (Figure 3e and Figure S3). Thus, it is conceivable that PcoB operates as a monomer, as also supported by the single molecule observed in the asymmetric unit of the crystal packing.

### 2.3 PcoB harbors a highly electronegative pore compatible with Cu uptake

**FIGURE 5:**
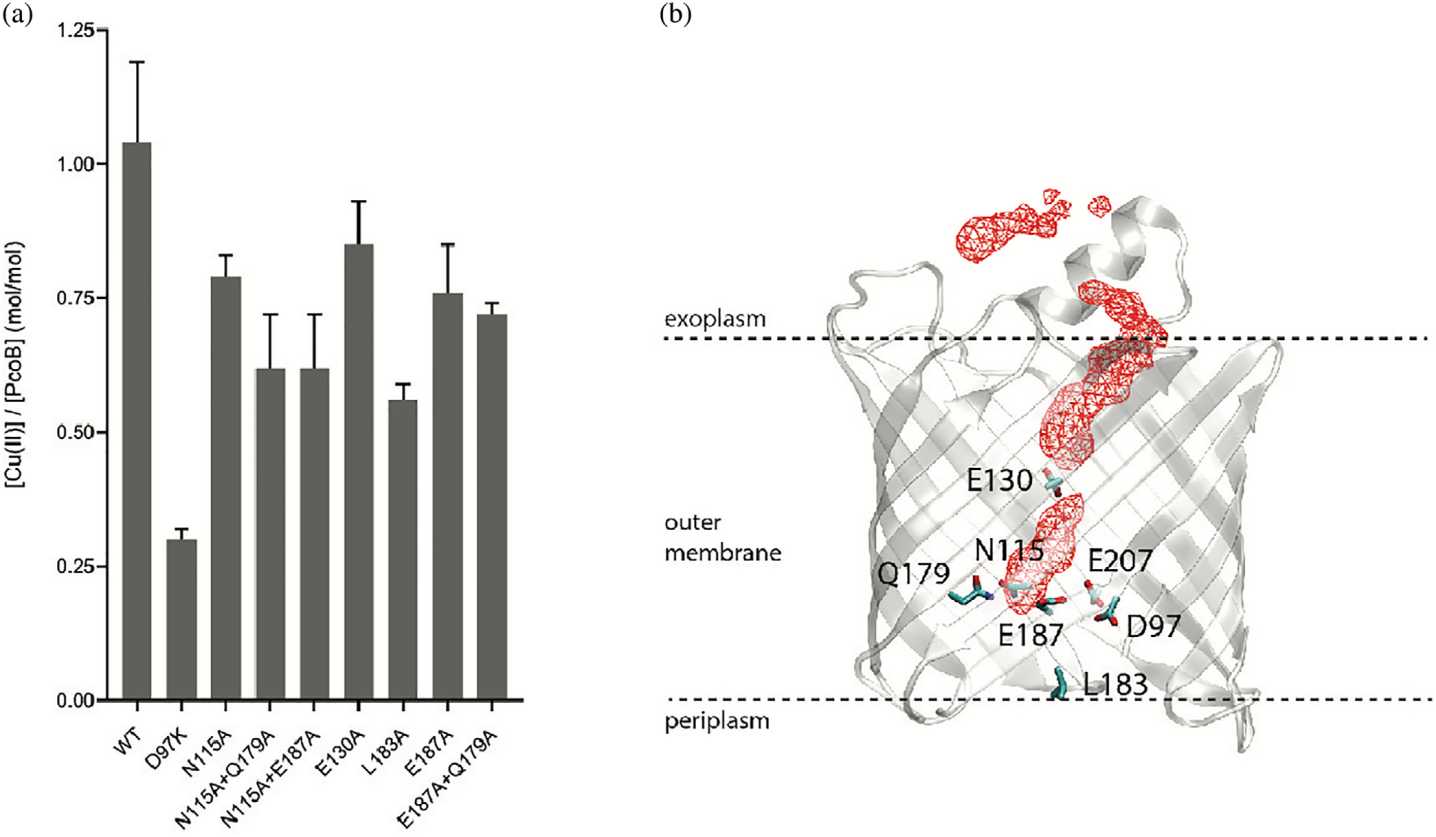
Restricted passage and copper binding. (a) Cu^2+^ binding stoichiometry of PcoB mutants as determined using ICP‐MS. Data denotes 3 independent measurements and error bars represent SD. The data is congruent with a single ion binding site located at the D97‐E207 constriction. (b) Isodensity surface (red) at 70% occupancy representing Cu^2+^ positions in the MD simulation to assess ion passage across PcoB

Strikingly, a 12 × 18 Å wide cavity is present on the extracellular side (Figure 4a, top right), as established by condensed loops between strands in the region leaving the aperture uncovered. From this region, a funnel‐like pore (Figure 4c–e and Figure S3A) is penetrating almost the entire barrel, becoming narrower towards the periplasm, with a remarkably electronegative funnel surface throughout (Figure 4a, bottom right). The outline of the pore is supported by the presence of numerous and continuous crystal waters (the maximum observed oxygen‐to‐oxygen distance is 4.4 Å) (Figure S4A). Along the pore, multiple negatively charged‐paired residues are present, in particular: E130‐E223 towards the extracellular side, D97‐E207 and N115‐Q179 towards the periplasm, which are both placed perpendicular to E187 (Figure 4c–e). The E130‐E223 and D97‐E207 pairs are both highly conserved among PcoB proteins, and form interacting carboxylic acid‐carboxylate hydrogen bonds that assist in flattening the barrel, and at the same time lines the pore (Figure S3). This negatively charged interior reminds of the outer membrane protein component, CusC, of the CusABC system (Figure 4b).^29^ The pore properties and the similarity to CusC are congruent with PcoB metal conductance as Cu^2+^ rather than Cu^+^_._ This is because, based on the HSAB Pearson theory,^35^ Cu^+^ typically rely on soft ligand coordination (Met and Cys) for ion transfer, while the Cu^2+^ is favored by funneling and potential coordination by harder ligands (such as Asp, Glu or His). The presence of these potential transient ligands in the pore hints at inward Cu^2+^ flux (from the surroundings), considering the highly electronegative surface on the extracellular side, presumably attracting divalent ions from the outside of cells, thereby supporting the role of PcoB as a Cu^2+^ importer. Based on the structure, it is also possible that other divalent ions such as Zn^2+^ are facilitated by PcoB, but this remains to be validated experimentally. However, the structure was obtained in Cu‐free conditions, and consequently there are no indications of the metal in the pore. Analogously, soaking and co‐crystallization experiments to identify Cu presence in the pore were fruitless. In contrast, inductively coupled plasma mass spectrometry (ICP‐MS) data on purified protein suggest Cu^2+^ is bound in the pore when metal has been supplemented to the sample as PcoB_FL_ and PcoB_Δ27−81_ each display one bound Cu^2+^ per molecule, likely representing a high‐affinity site, but additional weaker transient sites cannot be excluded as multiple residues capable of binding Cu are present (also in the N‐terminal tail) (Figure 3a). Corresponding analysis using ICP‐MS of the of the D97K mutant indicates a significantly impaired Cu^2+^ binding to PcoB (approximately 70% lower than PcoB_FL_) in agreement with the D97‐E207 being directly involved in high‐affinity Cu binding at the end of the funnel or that the anticipated salt‐bridge in the mutant prevents Cu passage, closing the pore (Figure 5 and Figure S4).

**FIGURE 6:**
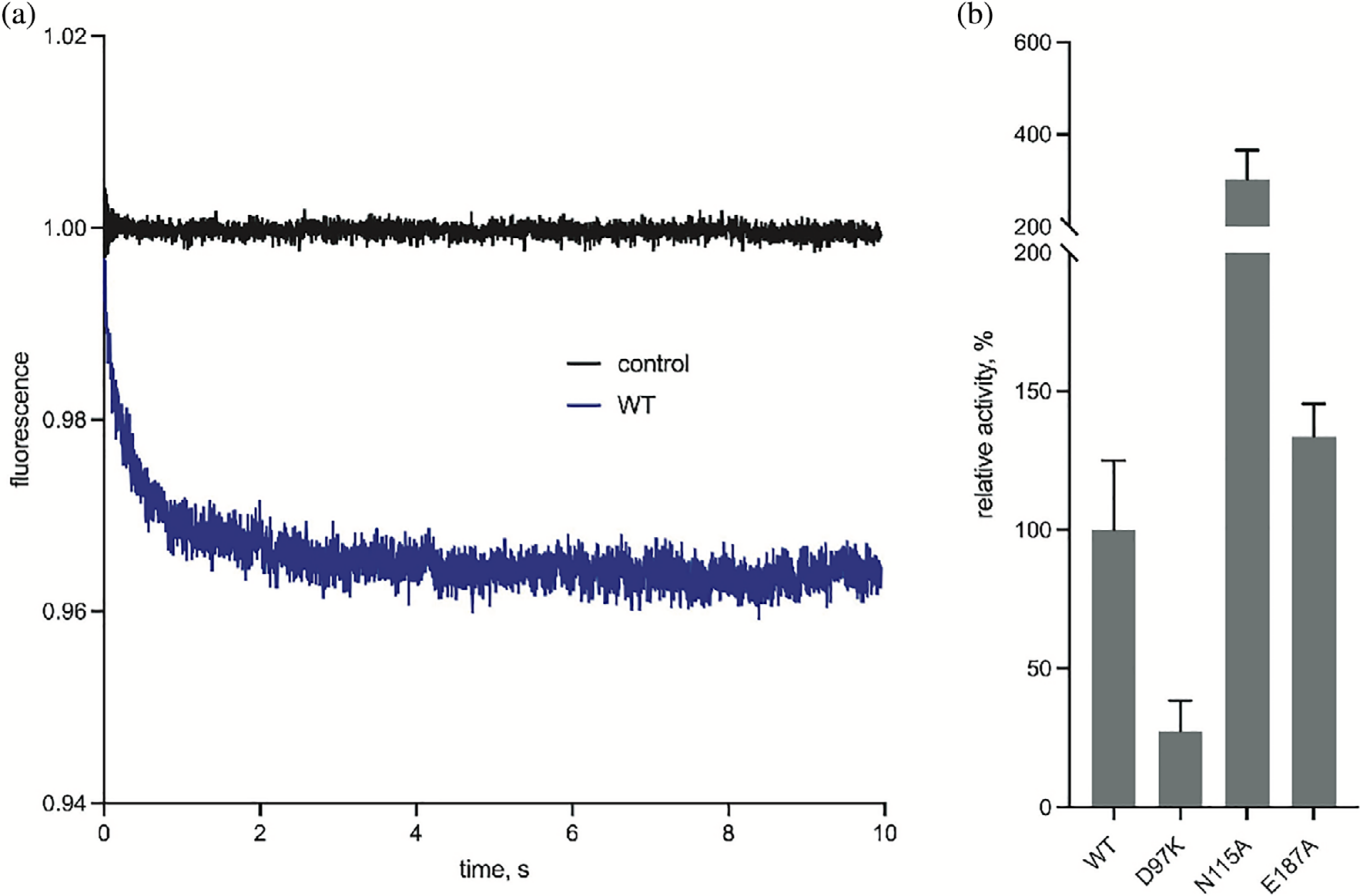
Functional characterization based on a proteoliposome assay. (a) Wild type PcoB (blue) demonstrates flux of Cu^2+^, clearly deviating from the control measurements (black) performed using empty liposomes. Traces originate from five runs based on triplicate reconstitutions. (b) Bar diagrams of the relative activity of the investigated PcoB mutants with wild type set as 100%. The activity has been compensated for the amount of protein incorporated into the liposomes (see Table S1 and Figure S5). The shown forms were all well‐behaved throughout the isolation and characterization process

In this light, we set out to investigate if PcoB indeed facilitates Cu^2+^ flux across cellular membranes. Utilizing a protocol developed for reconstitution and flux measurements of other porins,^36^ PcoB_FL_ and the D97K mutant were successfully reconstituted, and protein‐free liposomes employed as a as control (Figure 6). As evident from the averaged traces from three reconstitutions and compensation of differences in reconstitution efficiency of the different PcoB forms (Figure S5 and Table S1), wild type clearly conducts Cu^2+^ (Figure 6a), while the mutation appears less active than the wild type (Figure 6b), suggesting the central D97 represents a key feature for permitting Cu binding and flux.

### 2.4 The periplasmic constriction

In contrast to the extracellular side, the waterline of the pore becomes partially obstructed at the intersection between D97, E187, D209 and E207, with D209 and the adjacent Y184, being in proximity to the periplasm and yet capping the pathway together with L183 (Figure 4c and Figure S3). Through analysis of the water molecules in the region detected in the structure, two possible Cu^2+^ paths are revealed towards the periplasm (Figure 4c and Figure 3a), as also confirmed by surface analysis of the internal cavities. However, one of these is lined by several positive residues, most notably R250 and R293 in the immediate vicinity of D97‐E207, disfavoring involvement in Cu passage (Figure 4c). In contrast, the second path is rich in negatively charged residues (Figure 4c), and links directly to the electronegative periplasmic vestibule (Figure 4a) through a network consisting of N115, Q179 and E187 (Figure 4c–e). Notably, these three possible gate residues are well conserved (N115 is frequently replaced by other long‐side chain residues) and, in addition, analysis of evolutionary coupled pairs using the EVcouplings server (https://evcouplings.org/) suggest Q179 to be linked to N115, in agreement with a shared functional purpose (Figure S3).^37^ Alanine replacements of these residues had little to no effect on the binding stoichiometry as evaluated by ICP‐MS experiments (Figure 5). This may suggest these amino acids are rather important for the overall Cu conductance and potential transient Cu^2+^ binding when the pore is fully open, without establishment of discrete high‐affinity sites that would affect binding stoichiometry.

Considering we were unable to obtain a complementary structure in the presence of Cu, to further investigate how ions may bridge the restriction region, we instead generated alanine substitutions *in silico* of relevant residues to assess the effect on the local environment. Notably, mutations of L183, Y184, and D209 on the R250‐R293 pathway do not result in a significant pore widening (Figure S4C), despite the presence of crystal waters along this pathway. On the other hand, replacements of the residues of the complementary vestibule, with E187, N115 and Q179, resulted in a significant reduction of the constriction independent of mutation (Figure S4B). Notably, mutation of Q179 alone caused a substantial widening, hinting at the N115‐Q179‐E187 vestibule pathway playing an important role for the Cu^2+^ passage (Figure S4B). Next, unbiased molecular dynamics simulations of the recovered PcoB structure were exploited to shed further light on the ion passage. Congruent with our structural analysis, we observed that Cu^2+^ is not capable of passing through this constriction towards the periplasm (Figure 5b). Such behavior has however also been found for certain well‐established ion channels such as ClC‐1,^38^ where a narrow constriction region is bridged by ion‐transferring sidechains, representing a possible role of N115‐Q179‐E187 in PcoB.

We then reverted to our liposome assay to investigate wild type and mutant PcoB forms in vitro (Figure 6). Indeed, alanine substitution of N115 and E187 with the intention to widen the pore elevated the Cu^2+^ flux. The N115 form showed a dramatic three‐fold increase of the transfer rate, while E187A was augmented by 50%, congruent with a role in gating of the ion transfer. Two double mutations of the constriction region, N115A/Q179A and E187A/Q179A, however yielded an additional band on SDS‐PAGE following liposome reconstitution. We interpreted the response as a sign of instability and refrained from further analysis of these mutant forms (Table S1 and Figure S5A). It is possible that the shift of Q179 is responsible for the apparent degradation. Another mutant form in this region, D156K, demonstrated aggregation already in the size‐exclusion purification (Figure S6), perhaps due to the change of the local charge.

Analogously, alanine replacement of L183, a surface exposed residue that is capping the R250‐R293 pathway, also suffered from instability as detected by SDS‐PAGE. Y184A and D209K, the latter in immediate connection to R250, demonstrated a similar behavior as D156K, despite Y184 being in direct contact with the surrounding environment (data not shown). Thus, it is likely that L183, Y184 and D209 play a role in maintaining the local architecture rather than participating in ion flux (Table S1 and Figure S5A). The functional characterization was also complemented by ICP‐MS measurements of several of the mutant forms that were possible to recover in detergent form, however the metal binding stoichiometry remain largely unaffected for all forms (Figure 5). Thus, it is likely bridging between the extracellularly exposed funnel and the periplasm does not include ion bindings sites per se, rather a direct transfer via amino acid ligands. Aggregated, these findings point towards an expected sensitivity for mutations of the periplasmic‐facing region of PcoB. Furthermore, the data suggest ion transfer is achieved via the single Cu^2+^‐binding site in the entire protein, located at the very end of the electronegative funnel at D97‐E207 (mutation of which obstructs conductance). Next Cu^2+^ passage occurs through N115‐Q179‐E187 (with pathway enlarging single alanine substitutions elevating the flux), and finally, via the electronegative vestibule that is in direct contact with the periplasm, without presence of distinct check points equivalent to D97‐E187.

### 2.5 The *pco* operon may act as a defense mechanism in high Cu environments

The composition of the *pco* operon readily suggests its role in maintenance of periplasmic Cu levels: three of the core proteins are periplasmic proteins, responsible for detoxification (PcoA) and Cu binding (PcoC and E)^21^, ^22^ (Figure 1). Moreover, the two‐component system PcoRS senses periplasmic Cu levels and regulates the expression of the other proteins, except PcoE that is regulated by CusRS.^20^ Additionally, the *pco* plasmid has no effect in cells that are unable to export cytoplasmic Cu: deletion of the Cu^+^ exporting P‐type ATPase CopA abolishes the protective effect of *pco*,^8^ an observation that is in agreement with the notion that pco operon is a part of the periplasmic Cu defense mechanism.

The nature of the protection provided by the *pco* operon has remained unclear, yet the functions of three of the proteins are well‐established: PcoE being a Cu “sponge”, capable of rapid binding a large number of Cu ions,^21^ and PcoC being a Cu chaperone that binds both Cu^+^ and Cu^2+^, and delivers Cu^+^ to PcoA, a multicopper oxidase that catalyzes the oxidation of Cu^+^ to Cu^2+^, rendering Cu less toxic.^22^ In contrast, the specific roles of the membrane proteins PcoB and PcoD have been more elusive. PcoB, a classical outer membrane protein as shown in this work, was previously suggested to provide an efflux pathway for periplasmic Cu, presumably as the last step of a molecular defense system against high‐environmental stress. However, such efflux requires energy input, as the exported ion moves against a gradient: the extracellular environment contains more Cu than the periplasm when cells are exposed to Cu stress. The obtained structure in this work, demonstrating the presence of a highly electronegative pore compatible with cellular Cu^2+^ uptake, is thus consistent with PcoB acting as an outer membrane Cu^2+^ importer.

Cu export to the extracellular side is achieved in *E. coli* by the CusFCBA system (Figure 1, magenta), the architecture of which is however diametrically different than that of the *pco* system (Figure 1, cyan). CusA is an inner membrane protein that exploits the energy stored in the proton gradient to pump out Cu^+^ ions via a large tunnel consisting of CusB and CusC proteins, together spanning the entire periplasm and forming an exit pathway through the outer membrane.^12^ The direct coupling between the inner membrane, the periplasm, and the outer membrane allows for the energy‐requiring Cu^+^ export reaction to occur. Contrarily, the *pco* system does not contain proteins sufficiently large to form a direct contact to the inner membrane. While the Cu chaperone PcoC could potentially deliver Cu^2+^ to PcoB, which then becomes permeable upon PcoC binding, such scenario still does not provide a molecular basis to explain how energy input is obtained to guarantee Cu export against a concentration gradient. These arguments all support the notion of PcoB serving as an importer, although such a function may be counter‐intuitive for a defense system.

Upon environmental stress, the outer membrane becomes the first barrier in the defense. Nonspecific porins are downregulated and a number of selective outer membrane proteins are rather inserted in the membrane, to still ensure selective influx of necessary molecules and cofactors, yet preventing toxicity.^39^ It is possible to imagine a setup where Cu in the surroundings can be imported into the periplasm to acceptable levels (i.e., sufficient to metallize selected proteins, but not more), but in the presence of a periplasmic control system, ready to rapidly react to elevated concentrations of Cu. Expression of a Cu‐specific porin and downstream periplasmic Cu chaperones would allow for more control and quicker response.

Our structural, functional, and in vivo data corroborate the notion that PcoB play a role in such an unorthodox defense system. The structure is highly electronegative and harbors a considerable funnel freely accessible from the outside, clearly in agreement with attraction of charged matter in the surroundings (Figure 4). Nevertheless, PcoB is partially obstructed as detected by our structural analysis, suggesting a gradient may be necessary to allow passage. Indeed, Cu flux is permitted when a gradient is applied using the employed proteoliposome assay (Figure 6). Analogously, the complete PcoB system increases viability at elevated concentrations of Cu, although the morphology of the cells is altered, perhaps due to the increased levels of metal in the periplasmic space (Figure 2). In contrast, PcoB alone does not provide a similar molecular defense and instead increases the metal sensitivity, as the other components of the Pco assembly do not complex and potentially buffer the imported Cu. The potential roles of periplasmic Pco components in periplasmic copper buffering (PcoE) and detoxification (PcoA and PcoC) are consistent with the proposed role of PcoB, in agreement with the observation that PcoA can be found without PcoB in certain organisms, but not the opposite.^24^

Taken together, our findings improve our understanding how bacteria handle excess environmental Cu, likely importing Cu^2+^ through PcoB to the periplasm which may serve a dual purpose of providing essential metal at low‐Cu conditions and/or sequester environmental metals under Cu‐stress. In conditions of low‐Cu availability, this system would be capable of generating a dynamic pool of periplasmic Cu that can be possibly utilized for cellular import (by PcoB to the periplasm and perhaps also to the cytoplasm via PcoD). Thus, previous observations supporting the roles of the Pco operon in both resistance upon Cu stress, or acquisition upon Cu depletion are reconciled. The results also open for an attractive possibility to increase the Cu sensitivity in PcoB containing pathogens through blockage of the electronegative funnel from the outside of the cells, as a means to combat infections.

## 3 MATERIALS AND METHODS

### 3.1 In vivo Cu tolerance assays

For the agar plate study, bacterial BL21(DE3) cells were transformed with plasmid pET‐22b‐PcoB and grown on LB‐agar plates supplied with 50 μg/ml ampicillin for 16 h at 37°C (these conditions were used for all Cu tolerance growths unless otherwise is stated). Cells harboring empty vector pET‐22b were used as control. A single colony was inoculated in 5 ml LB culture for approximately 8 h until the optical density at 600 nm (OD_600 nm_) reached 0.8. The cells were spin down, washed and resuspended in fresh LB media to OD_600 nm_ = 0.1, and then diluted in 10‐fold increments using LB media. The 5 μl drops were spotted onto the LB‐agar plates containing defined amounts of CuCl_2_ (0 and 1 mM), and with the media pH‐adjusted to 7.0 (following supplementation of CuCl_2_). The plates were incubated at 37°C for 16 h to compare the growth of the colonies.

For the electron microscopy study, the transformed cells were first cultured in 5 ml LB to OD_600 nm_ = 0.6, and then the cells were cultured for 16 h with starting OD_600 nm_ = 0.05 in 5 ml containing the desired CuCl_2_ concentration (0 and 2 mM), with the pH of the media adjusted to 7.0 (following supplementation of CuCl_2_). The cultured cells were pelleted using 6,000 ×*g* for 10 min and then washed three times using PBS buffer to remove dead cells. Pellets of bacteria were fixed with 2% v/v glutaraldehyde in 0.05 M sodium phosphate buffer (pH = 7.2). The pellets were embedded in Agarose, rinsed three times in 0.15 M sodium phosphate buffer (pH = 7.2), and subsequently postfixed in 1% w/v OsO_4_ with 0.05 M K_3_Fe(CN)_6_ in 0.12 M sodium phosphate buffer (pH = 7.2) for 2 h. The specimens were dehydrated in graded series of ethanol, transferred to propylene oxide and embedded in Epon according to standard procedures. Sections, approximately 60 nm thick, were cut with a Ultracut 7 (Leica, Wienna, Austria) and collected on Cu grids with Formvar supporting membranes, stained with uranyl acetate and lead citrate, and subsequently examined with a Philips CM 100 Transmission EM (Philips, Eindhoven, The Netherlands), operated at an accelerating voltage of 80 kV. Digital images were recorded with an OSIS Veleta digital slow scan 2 × 2 k CCD camera and the ITEM software package.

### 3.2 Expression and purification of PcoB

The gene coding for *E. coli* PcoB including its signal peptide (UniProt Accession No. Q47453) was codon optimized and synthesized by Genscript. A 6 × His tag followed by a TEV cleavage site was introduced between S26 and V27 by overlap PCR to facilitate protein purification. The N‐terminus was truncated using PCR, thereby removing 55 residues (27–81) to generate PcoB_Δ27−81_ Next, the full‐length PcoB and PcoB_Δ27−81_ were cloned into the pET‐22b expression vector using *Nde*I and *Xho*I cleavage sites. The expression plasmids were transformed into the C43 (DE3) *E. coli* strain. Single colonies were incubated at 30°C for 16 h in 5 ml LB medium supplemented with 100 mg/ml ampicillin. The preculture was transferred into 1 L LB cultures supplied with 100 mg/ml ampicillin and cultivated at 30°C with shaking at 180 rpm. Protein expression was induced at 25°C for 16 h with final concentration of 0.5 mM Isopropyl β‐D‐1‐thiogalactopyranoside (IPTG) when the OD_600_ = 0.6.

To solve the crystallographic phase problem via selenomethionine (SeMet) phasing, three mutants (L93M, L146M and L213M) were introduced to facilitate the SeMet labelling, yielding PcoB_Δ27−81,3 × L/M_. The *E. coli* 834(DE3) strain and SelenoMet™ medium (Molecular Dimensions Limited) were used for the SeMet labelling protein expression. The PcoB_Δ27−81,3 × L/M_ plasmid was transformed into the *E. coli* 834(DE3) strain (a gift from LP3) and single colonies were inoculated in 5 ml LB medium supplemented with 100 mg/ml ampicillin at 37°C for 8 h. The cells were pelleted and washed three times in 1 ml of sterile water, resuspended in 1 ml of sterile water and cultured at 37°C for 16 h in 100 ml minimal media containing L‐methionine. Next, the cells were pelleted and washed three times in 100 ml sterile water, resuspended in 1 ml water, transferred into 1 L minimal media containing L‐SeMet, cultured for 2 h at 30°C, and then induced with 1 mM IPTG at 30°C for 6 h.

The cells were harvested by centrifugation at 8000×*g*, suspended in Tris buffer (20 mM Tris, pH = 8, 500 mM NaCl, 10% v/v Glycerol) and disrupted using sonication. The cell lysate was centrifuged at 165,000×*g* in a Beckman ultracentrifuge (45 Ti rotor, Beckman) for 1 h and the membrane fraction was resuspended in the same Tris buffer containing 1% w/v N‐laurysaccide, and then stirred at 18°C for 1 h. Subsequently, the outer membrane was pelleted by ultracentrifugation at 165,000×*g* for 1 h. The outer membrane pellets were solubilized in Tris buffer containing 2% Elugent (Calbiochem) at 4°C for 16 h with stirring. The extract was centrifuged at 190,000×*g* for 30 min and the supernatant was loaded to 5 ml His‐trap column (Cytiva) equilibrated with 30 ml Tris buffer containing 0.05% w/v lauryldimethylamine N‐oxide (LDAO). Then, the column was washed with six column volume (CV) equilibration buffer containing 50 mM imidazole. The protein was eluted with equilibration buffer containing 300 mM imidazole. The eluted protein fractions were pooled, concentrated and buffer exchanged using Ultra‐15 centrifugal concentrators (Amicon) with 50 kDa MW cut‐off to remove excess imidazole. Next, TEV protease was added with a molar ratio of 1:10 to remove the His tag through incubation at 4°C for 16 h. Subsequently, the cleaved sample was loaded to a preequilibrated 5 ml His‐trap column using equilibration buffer. The tag removed protein sample was collected in flow‐through and eluted with equilibration buffer containing 40 mM imidazole. Desired fractions were concentrated, and buffer exchanged using 50 kDa MW cut‐off concentrators (Amicon) to SEC buffer 20 mM Tris, pH = 8.0, 100 mM NaCl, 5% w/w Glycerol and 0.4% w/w C_8_E_4_ detergent. As a polishing step, the protein was finally SEC purified (Superdex 200 10/300; GE Healthcare) and the purified protein was concentrated to 10 mg/ml for the crystallization.

### 3.3 Crystallization

Initial crystallization for native PcoB and SeMet‐PcoB was performed using MemGold, MemGold2, Memstar and MemSys screens from Molecular Dimensions by sitting‐drop vapor diffusion using a Mosquito robot at the Lund protein production platform (LP3) by mixing 0.2 μl protein sample with 0.2 μl reservoir solution. The initial hits appeared following 2 days in a condition with 8% w/v PEG4000, 0.4 M NaSCN, 0.1 M sodium acetate, pH = 4.0, and was optimized using grid‐screening and using larger hanging‐drop vapor diffusion drops. The best native PcoB crystals grew in 10% w/v PEG4000, 0.8 M NaSCN, 0.1 M sodium acetate pH = 4.0. SeMet‐PcoB crystals were obtained from in 30% w/v PEG400 and 0.1 M sodium citrate, pH = 4.0. The crystals were cryoprotected, harvested and flash‐cooled in liquid nitrogen for data collection at Swiss Light Source (SLS).

### 3.4 Data collection and structure determination

Native and SeMet PcoB X‐ray diffraction data was collected at the Paul Scherrer Institute, Switzerland on beam line X06SA (SLS). The data was processed and scaled using the software XDS.^40^ The crystals belonged to space group C2221 with cell parameters a = 64.489 Å, b = 75.538 Å, c = 91.51 Å, α = β = γ = 90°. The SeMet data was collected at the wavelength 0.9798 Å. Initial crystallographic phases were calculated by Phenix AutoSol using SAD phasing.^41^ Seven selenium atoms were located and refined, then Autobuild^42^ was performed to generate the initial model. The initial structure was employed as starting model to determine the PcoB structure of high‐resolution native dataset. Model building was performed manually in Coot^43^ and refined by phenix.refine^44^ in iterative steps. The 198 residues were built and refined in the final structure with 95.85% of residues in the favored region of the Ramachandran plot (Table 1). The final model displayed Ramachandran favored, allowed and outliers of 95.88%, 4.12%, and 0.00%, respectively. Rotamer outliers were 0.00% and the clash‐score was 3.72.

### 3.5 Cu^**2**+^ binding stoichiometry determination with ICP‐MS

Cu^**2**+^ binding stoichiometry to wild type PcoB and mutants was measured by ICP‐MS. The protein forms, purified as mentioned above, were diluted to 3–10 μM with buffer containing 50 mM Tris, pH = 8.0, 500 mM NaCl, 10% w/v glycerol, and 0.05% w/v LDAO, or 20 mM Tris, pH = 8.0, 500 mM NaCl, 10% w/v glycerol, and 0.8% w/v OG. 2–5equivalents (mol:mol)  of CuCl_2_ were subsequently added to the protein solution and incubated at 18°C for 15 min and excess Cu^2+^ was removed with 5 ml HiTrap desalting columns (Cytiva) packed with Sephadex G‐25 resin, preequilibrated with the respective buffer. Following elution, the protein concentration was determined by a Bradford assay using BSA as standard. For ICP‐MS sample preparation, eluted protein samples were digested in 8% v/v HNO_3_ at 80°C for 12 h. Samples were subsequently diluted to adjust the HNO_3_ concentration to 3% and ICP‐MS was performed with a Hewlett‐Packard 4,500 ICP mass spectrometer (Agilent Technologies) connected to a CETACASX‐500 auto‐sampler for sample injection. Control experiments were conducted in the same buffer excluding the protein for background correction. For the binding experiment high‐purity TraceSelect nitric acid, H_2_O and ICP‐MS standards were purchased from Sigma‐Aldrich.

### 3.6 Proteoliposome Cu^2+^ flux assay

PcoB forms were evaluated functionally in liposomes referred to as small unilamellar vesicles (SUVs). Following purification, wild type PcoB and different mutants were reconstituted in liposomes with a lipid composition of *E. coli* polar lipids (Avanti, US) and 1‐palmitoyl‐2‐oleoyl‐sn‐glycero‐3‐phosphocholine (POPC) (Sigma Aldrich) in a 3:1  (mol:mol) ratio. The lipids were dissolved in chloroform in a glass vial to a concentration of 25 mg/ml followed by treatment using N_2_ to form a thin lipid bilayer. The lipid film was kept under a N_2_ stream for 2 h to achieve complete chloroform removal. The lipid film was rehydrated with reconstitution buffer (20 mM Tris, pH = 7.4, 200 mM NaCl) with added fluorophore, 10 mM Pyranine (Sigma), to a concentration of 20 mg/ml lipids. The lipid suspension was applied to a sonication bath for three times × 15 min, with a 5 min break in‐between the cycles. Next, the lipids were frozen in liquid nitrogen and thawed three times, and then the lipids were passed through a 100 nm polycarbonate filter 11 times, using an extruder (Mini‐Extruder, Avanti). The lipids were diluted to 4 mg/ml with reconstitution buffer containing 25% v/v glycerol and 1% w/v OG, followed by gradual addition of 0.2% w/v Triton X‐100. PcoB and mutants were added to each their lipid suspension using a lipid‐to‐protein‐ratio (LPR) of 20 and each sample was dialyzed for 16 h at 4°C in reconstitution buffer. The samples were centrifuged at 57,000×*g* for 1.5 h and the resulting pellets were suspended in reconstitution buffer. Traditionally, Cu^2+^ flux assays are measured using fluorophores such as Fluozin‐1 or ‐3, which are being activated by addition of Zn^2+^ and quenched by addition of Cu^2+^. However, upon addition of Zn^2+^ and Cu^2+^ the PcoB protein appeared to degrade, in contrast to supplementation of Cu^2+^ only, where only a single band is present on SDS‐PAGE analysis. Consequently, we employed fluorescent dye pyranine, which has previously proven effective in measuring the flux of Cu^2+^ ions.^45^, ^46^, ^47^ The assay was performed on an SX‐20 Stopped‐Flow Spectrometer system (Applied Photophysics) equipped with a 495 bandpass filter, where the liposomes were mixed with reaction buffer with 0.1 mM CuCl_2_. Data were collected at 510 nm at a 90° angle for 10 s. All data were collected at 18°C. Empty liposomes were used as a negative control. The data were analyzed and plotted in Pro‐Data Viewer (Applied Photosystems). Data for each sample were the average of five readings. Data were fitted using a double exponential fit. The smallest rate constant is unaffected by changes in PcoB reconstitution efficiency, while the larger rate constant corresponds liposomes containing PcoB. The rates where then adjusted (Equation 1) using the wild type experiments (equivalent to 100% activity), to obtain the relative activity a_rel_. k_exp_ is the k‐rate obtained from a single experimental reconstitution, k_WTavg_ is the three k‐rates obtained from the WT experiments, then averaged. Control experiments on empty liposomes yielded a flat‐line, and thus a k‐rate of 0 (k_control_). The reconstitution experiments were performed three times to achieve data reproducibility.

(1) $$a_{\mathrm{rel}}=\frac{k_{\exp}-k_{\text{control}}}{k_{\text{WTavg}}}∙100\%=\frac{k_{\exp}∙100}{k_{\text{WTavg}}}.$$

### 3.7 MD simulation system design and analyses

The asymmetric lipid bilayer was built using the membrane builder^48^ in CHARMM‐GUI^49^ with the inner leaflet containing 1,1′‐palmitoyl‐2,2′‐vacenoyl cardiolipin (PVCL2), 1‐palmitoyl(16:0)‐2‐palmitoleoyl(16:1 cis‐9)‐phosphatidylethanolamine (PPPE) and 1‐palmitoyl(16:0)‐2‐vacenoyl(18:1 cis‐11)‐phosphatidylglycerol (PVPG) lipids while the outer leaflet contained lipopolysaccharides (LPS) with lipid A of type 1 tail and core R1 and a 1 o‐antigen polysaccharide chain.^50^ protein was inserted into the membrane based on prediction from the Orientations of Proteins in Membranes (OPM).^51^ Predicted pKa values were calculated with the Propka‐3.1 program^52^ and residues E130, E187, E207, and E243 were protonated. The system was simulated using the GROMACS 2019 MD simulation engine^53^ with the CHARMM36 all‐atom force field.^54^, ^55^ The system contained 25,465 TIP3P water molecules, 550 Na‐ions and 47 Cl‐ions (in total 131,956 atoms). A 5,000‐step energy minimization was followed by a 30 ns equilibration run during which the protein backbone, side chain, lipid, and water atoms were successively unrestrained in six consecutive 5‐ns steps leading to the 500 ns production run. The simulation time step was 2 fs and a Parrinello‐Rahman semi‐isotropic pressure coupling^56^, ^57^ with a compressibility of 4.5e‐5 bar^−1^ was applied with a coupling constant of 1.0 at 1 bar and the temperature was maintained at 303.15 K using the Nose‐Hoover temperature coupling.^58^, ^59^ A Cu^2+^ ion was added to the final frame of the production run of 500 ns by replacing a Na^+^ ions at the outer membrane entrance and reparametrizing according to Cu^2+^ parameters from.^60^ The Cu^2+^ ion was pulled from the outer to inner leaflet using the Gromacs pull code. The Cu^2+^ ion was pulled for 3.5 ns at a pulling rate of 1.5 nm/ns at a harmonic force of 1,000 kJ/mol/nm^2^. The Na and Cl‐ions were restrained during pulling to avoid interference with ion‐entry dynamics.

## AUTHOR CONTRIBUTIONS

**Ping Li:** Conceptualization (lead); data curation (lead); formal analysis (lead); investigation (lead); methodology (equal); validation (supporting); visualization (supporting); writing – original draft (equal); writing – review and editing (equal). **Niloofar Nayeri:** Data curation (equal); formal analysis (equal); investigation (equal); validation (supporting); visualization (equal); writing – original draft (equal); writing – review and editing (supporting). **Kamil Górecki:** Formal analysis (supporting); investigation (supporting); validation (supporting); visualization (supporting); writing – review and editing (supporting). **Eva Ramos Becares:** Formal analysis (supporting); investigation (supporting); writing – review and editing (supporting). **Kaituo Wang:** Formal analysis (supporting); investigation (supporting); supervision (supporting); validation (supporting); writing – review and editing (supporting). **Dhani Ram Mahato:** Data curation (supporting); formal analysis (supporting); investigation (supporting); writing – review and editing (supporting). **Magnus Andersson:** Formal analysis (supporting); funding acquisition (supporting); investigation (supporting); resources (supporting); supervision (supporting); visualization (supporting); writing – review and editing (supporting). **Sameera S. Abeyrathna:** Data curation (supporting); formal analysis (supporting); investigation (supporting); writing – review and editing (supporting). **Karin Lindkvist‐Petersson:** Formal analysis (supporting); funding acquisition (supporting); investigation (supporting); resources (supporting); supervision (supporting); writing – review and editing (supporting). **Gabriele Meloni:** Formal analysis (supporting); funding acquisition (supporting); investigation (supporting); methodology (supporting); supervision (supporting); validation (supporting); visualization (supporting); writing – review and editing (supporting). **Julie Winkel Missel:** Formal analysis (supporting); investigation (supporting); supervision (supporting); validation (supporting); visualization (equal); writing – original draft (equal); writing – review and editing (equal). **Pontus Gourdon:** Conceptualization (equal); data curation (supporting); formal analysis (supporting); funding acquisition (lead); investigation (lead); methodology (supporting); project administration (lead); resources (lead); supervision (lead); validation (supporting); visualization (supporting); writing – original draft (lead); writing – review and editing (lead).

## Supporting information


**Appendix S1** Supporting information

## Data Availability

The structure of PcoB_Δ27−81_ reported in this paper will be released by the Protein Data Bank upon acceptance of this manuscript (PDB, PDB‐ID 7PGE).
