# Supplementary material for: PcoB is a defense outer membrane protein that facilitates cellular uptake of copper
Source: Protein Sci. 2022 Jun 21;31(7):e4364. doi: 10.1002/pro.4364 (PMC9210255; doi:10.1002/pro.4364)
Supplement: Supplementary file 1 — Appendix S1 Supporting information [file PRO-31-e4364-s001.docx]

**Supplementary Figure 1. Design of PcoB. (A)** Schematic overview of the PcoB construct design. **(B)** Alphafold model (yellow and red) of full-length PcoB (cyan) in overlay with the crystal structure of PcoB. Red denotes low confidence of the AlphaFold prediction (30, 31).

**Supplementary Figure 2. Details of the structure of PcoB.** The disordered loop region in the PcoB structure (cyan). The final 2Fo-Fc electron density is shown in blue mesh, σ = 1.0. Arrow points to the unmodeled loop (dashed line).

**Supplementary Figure 3. Sequence conservation among PcoB proteins.** Accession numbers refer to Uniprot with *E. coli* PcoB highlighted in bold. Red columns indicate the most conserved residues. Structurally and functionally important residues are shown in purple with the residue number of *E. coli* PcoB indicated above each row. The location of the β-strands of the structure is shown. The alignment was generated through a Uniprot Blastp search using the *E. coli* PcoB as a template, thereby securing 250 proteins. These sequences were aligned using Clustal Omega and sequence redundancy (higher than 75 % identity) was removed using Jalview.

**Supplementary Figure 4. Two putative exit paths in PcoB.** **(A)** The structurally determined wild-type PcoB (gray) does not provide a continuous pore as shown using the surface of internal cavities (grey) and crystal waters (red spheres). *In silico* analysis was conducted to assess if mutant forms may render the pore more open. **(B)** Mutations related to the proposed exit pathway (orange). From left to right: E187A; E187A and N115A; Q179A. **(C)** From left to right: Y184A; Y184A and D209A; L183A; L183A and Y184A.

**Supplementary Figure 5. SDS-PAGE gels for various PcoB mutants.** **(A)** SDS-PAGE used for analyses of all PcoB forms reconstituted into liposomes (Supplementary Table 1). All mutations were quantified against wild-type (WT). The double mutants and L183A showed degrees of degradation, and were excluded from the final analysis. **(B)** Liposomal traces of empty control liposomes (black), Wild-type PcoB (blue), mutants N115A (red), E187A (yellow) and D97K (green). Traces originate from 5 runs based on triplicate reconstitutions.

**Supplementary Figure 6. Size-exclusion chromatography profiles of the studied PcoB forms.** His-tagged cleaved samples were injected into an pre-equilibrated Superdex 200 Increase 10/300 GL column mounted on an Äkta Avant system. The employed buffer included 20 mM Tris-HCl pH=8, 100 mM NaCl, 5 % Glycerol, 0.8% OG and the flow rate was 0.4 mL/min for all forms.

**Supplementary Table 1. Quantification constants of the three separate reconstitutions using ImageJ of the SDS-PAGE.** SDS-PAGE quantification analyses of all PcoB forms reconstituted in liposomes. All mutations were quantified against the first wild-type (WT) lane (Supplementary Figure 4) normalized to 1.0. The analysis was performed by plotting the band intensities in ImageJ and taking the integral from these separate plots. The factors were then divided by the integral from the WT lane 1, giving the constants in this table, of which the relative activity could be adjusted (Figure 6B).

|  | *WT* | *D97K* | *N115A* | *E187A* |
| --- | --- | --- | --- | --- |
| *constant #* |  |  |  |  |
| 1 | 1.0 | 2.2 | 0.7 | 1.0 |
| 2 | 1.1 | 2.2 | 1.6 | 0.8 |
| 3 | 1.1 | 2.2 | 0.9 | 1.5 |
